# Psychological distress and anxiety in Arab refugees and migrants during the COVID-19 pandemic in Germany

**DOI:** 10.1177/13634615221122536

**Published:** 2022-09-25

**Authors:** Jinan Abi Jumaa, Antonia Bendau, Andreas Ströhle, Andreas Heinz, Felix Betzler, Moritz Bruno Petzold

**Affiliations:** 1 Psychiatry and Psychotherapy, 14903 Charité Universitätsmedizin Berlin Campus Charite Mitte, Germany

**Keywords:** inequity, public mental health, psychological health, SARS-CoV-2 mental health

## Abstract

The COVID-19 pandemic is associated with various psychological stressors due to health-related, social, economic, and individual consequences, especially for minority groups such as refugees and other migrants who live in unstable conditions and have lost their social support groups. The aim of this study was to explore the impacts of the COVID-19 pandemic on this specific population in Germany.This study used a mixed-method approach. A total of 85 migrants took part in an online survey in Germany from April to July 2020. The questionnaire included demographic information and measures of psychological distress, anxiety and depressive symptoms, as well as risk and protective factors for psychological health during the COVID-19 pandemic. Semi-structured interviews with 10 refugees were conducted between May and June 2020. In our sample, 54.5% expressed fear of being infected with COVID-19. Participants spent several hours per day thinking about COVID-19 (*M*  *=*  3.13 hours). Psychological and social determinants of mental health showed stronger associations with anxiety regarding COVID-19 than experiences with the disease. Interviews showed that especially for refugees with limited information regarding access to medical treatment, the pandemic increased already-existing psychological symptoms and worries about their families back home and reminded them of their flight from their home country to Europe. The COVID-19 pandemic is associated with psychological distress, anxiety, and depression in refugees and migrants in Germany. Information on where to get medical treatment, if needed, is of utmost importance to this population group, in addition to other strategies such as maintaining a healthy lifestyle and social contacts, and acceptance of strategies to cope with anxiety and negative emotions.

## Introduction and background

In December 2019, a local outbreak of pneumonia-like symptoms of a new disease (COVID-19) caused by an unknown virus (SARS-CoV-2) was detected in Wuhan city, China (Dong et al., 2020). This local outbreak soon turned into a global pandemic, with 505,817,953 confirmed cases of COVID-19 worldwide, including 6,213,876 deaths as of April 2022 (World Health Organization (WHO), 2022). In Germany, at that point, the number of cases reached 25,130,137 confirmed cases with 136,125 deaths (WHO, 2022).

The World Health Organization (WHO) promoted a series of preventive measures to protect individuals from being infected with COVID-19 (WHO, 2020c). However, not all people have been able to abide by these safety measures. In particular, displaced populations like migrants, and specifically refugees who are often stigmatized, may have limited access to the healthcare system and often experience crowded substandard living conditions with limited access to safe water, sanitation, and nutrition. There thus may be an increased risk of refugees being infected with the virus (Lau et al., 2020). In some cases, refugees may work in settings that increase their risk of exposure. In Quebec (Canada), for example, refugees and asylum seekers who work in the healthcare sector are on the frontlines, working overtime and risking their lives to save the lives of others (Dalexis & Cénat, 2020). In addition to the psychological distress that comes with the pandemic, they face the anxiety of potential deportation when the crisis is over and continue to be confronted with racism and discrimination from the media and the community due to their asylum status (Dalexis & Cénat, 2020).

The WHO recognizes the importance of addressing the needs of refugees and migrants when responding to the COVID-19 pandemic (WHO, 2020b). The WHO also confirms that public health responses must consider health risks associated with movement, overcrowding, lack of nutrition, physical and mental stress and deprivation due to lack of housing or legal status among refugees and migrants (WHO, 2020a).

Apart from its physical effects, the COVID-19 pandemic has had several kinds of psychological and social impacts (Takieddine & Al Tabbah, 2020). A study in China that aimed to investigate if psychiatric patients experience more symptoms during the COVID-19 pandemic showed that the levels of anxiety, depression, and stress were higher in psychiatric patients than in healthy controls. This same clinical population was also found to exhibit worries about physical health, anger, impulsivity and intense suicidal ideation (Hao et al., 2020). Other studies in Germany showed that COVID-19 fundamentally impacts people’s anxiety and psychological distress levels (Liu & Heinz, 2020; Petzold et al., 2020). Petzold and colleagues (2020) found that almost half of their sample had fears regarding the consequences of the pandemic and spent large amounts of time per day thinking about COVID-19. A study conducted in Spain which aimed at assessing the psychological impact of the pandemic in a sample of the Spanish general population using Patient Health Questionnaire-2 and Generalized Anxiety Disorder Scale-2 found that 18.7% of the sample of 3,480 had a possible diagnosis of depression and 21.6% were likely to be diagnosed with anxiety (González-Sanguino et al., 2020). Discrimination and loneliness were related to a stronger psychological impact, while a sense of belonging, well-being, and self-compassion were protective (González-Sanguino et al., 2020). Likewise, a similar study in Spain of the psychological impacts of the pandemic found that more than a quarter of participants reported symptoms of depression, anxiety, and stress; and as the time spent in lockdown progressed, psychological symptoms increased (Ozamiz-Etxebarria et al., 2020).

In Italy, Mazza and colleagues (2020) found that quarantine had increased psychological distress among the Italian population. Being female and having an acquaintance infected were associated with increased levels of depression and stress; a history of stressful situations and medical problems was associated with higher levels of depression and anxiety. Killgore and colleagues (2020) investigated factors that might contribute to higher psychological resilience during the lockdown in the U.S. They found that resilience was greater among those who tended to go outside more often, who exercised more, who perceived more social support from family, friends, and significant others, who slept better, and who prayed more often.

There is evidence that refugees and other migrants may be at greater risk for stress related to the pandemic. Qiu and colleagues (2020) found that females, elderly people, people with higher education, and migrant workers among all occupations experienced the highest level of stress in a cross-sectional sample with 52,730 participants from the Chinese general population. The authors concluded that migrants might be a vulnerable population who have specific concerns and worries about viral exposure in public transportation when returning to work, and about delays in work time and consequent decrease in their income—all factors that may explain their high stress levels (Qiu et al., 2020).

The COVID-19 pandemic represents a source of stress due to uncertainty and lack of knowledge. Although refugees and migrants are among the groups likely to be the hardest hit, research on the psychological consequences of the pandemic on this population is limited, thereby increasing the likelihood that this population will be further neglected (Bhopal, 2020). According to demographic statistics from the Federal Office for Migration and Refugees (*Bundesamt für Migration und Flüchtlinge*; BAMF), the largest group of immigrants in Germany is from Syria (BAMF, 2019).

In light of these knowledge gaps, this study aimed to quantitatively assess the psychological distress (anxiety and depression) of Arab refugees and Arab-speaking migrants in Germany during the pandemic. It also qualitatively describes the experiences of refugees during the COVID-19 pandemic.

## Methods

This is a mixed methods study with an online survey and individual open-ended interviews of Arabic-speaking refugees in Germany. A mixed methodology for collecting and analyzing data is vital for understanding ‘hard-to-measure’ constructs and for confirming the accuracy of findings (Bowers et al., 2013). Mixed methods can balance the need for generalizability and sensitivity for specific perspectives when investigating minorities’ emotional distress, even in smaller samples (Nguyen et al., 2020). In this research project, the Arabic version of the questionnaire was available online in April 2020. It addressed different areas related to the general experiences of refugees during lockdown. The initial results of the questionnaire informed the development of the qualitative interview guide, which was used to get a more in-depth view on the specific problems and mechanisms and to better understand the refugees’ past experiences (flight and journey to Germany) as well as their daily experiences during the pandemic. The interviews were conducted between May and June 2020. The participants also had access to the questionnaire, but since the questionnaire was anonymous it was not possible to verify the identity of those who completed it.

### Instruments

An online questionnaire was designed by the anxiety disorders research group at the Charité University Medicine Berlin. A detailed description of the questionnaire can be found in recent publications (Bendau et al., 2020; Petzold et al., 2020). The questionnaire contained questions concerning demographic information, such as age, sex, and highest level of education. Other measures included: perceived risk of being infected within the next month, rated from 0% to 100%; daily average number of hours spent thinking about COVID-19; and hours spent on news media about COVID-19. The Patient Health Questionnaire-4 (PHQ-4) was used to screen for depressive symptoms (Kroenke et al., 2009). To assess selected aspects of anxiety regarding COVID-19, nine items were included (e.g., fear of being infected and fear of social or economic consequences), statements were rated on a 6-point Likert scale, ranging from 1 (“strongly disagree”) to 6 (“strongly agree”). In addition, the Covid-19-Anxiety Questionnaire (C-19-A), a modified version of the validated DSM-5 Severity-Measure-For-Specific-Phobia-Adult-Scale, was also used (Craske et al., 2013; Petzold et al., 2020).

The questionnaire was translated into Arabic by the first author; a blind back-translation was then performed by another Arabic speaker in the team. The questionnaire was then distributed online on Facebook groups (such as Place4Refugees, Refugees in Berlin) among Arabic-speaking refugees and migrants in Germany and through connections with the first author from previous fieldwork. All migrants included were not born in Germany but rather migrated from their home countries between 1985 and 2020.

### Recruitment and data collection

Recruitment was done via social media (Facebook groups and WhatsApp); no paid advertising was used. The questionnaire was accessible via the platform SoSci Survey and participants were directed toward the questionnaire by posting the survey invitation on social media platforms. The minimum age of participation was 18 years; participants had to be Arabic speakers, and either a refugee or migrant, and they also had to reside in Germany. There were no other inclusion or exclusion criteria.

The online questionnaire included 46 questions in total. Completing the entire questionnaire required 20–25 min. Data collection occurred from April 17, 2020 to July 16, 2020, after approval by the Ethical Review Committee of Charité – University Medicine Berlin (application number: EA1/071/20). All participants agreed to participate and signed an informed consent prior to the initiation of the survey.

### Statistical analysis

All analyses were carried out using IBM SPSS Statistics Version 26. For the analysis, mainly descriptive statistics and Pearson's correlations (significance level was set to .05 two-tailed) were used. Missing data was handled by pairwise deletion, which is a method in which data for a variable relevant to a specific assessment are included, even if values for the same individual on other variables are missing This is a commonly used method which has several disadvantages but is considered an easy-to-follow and transparent option in data sets with small amounts of missing data (Enders, 2022).

### Qualitative interview

A guide was developed with questions on topics to be discussed during the interviews. The main questions were: 1) What do you know about the novel Coronavirus? 2) How did you feel when you first knew about the novel Coronavirus? 3) How would you compare the COVID-19 anxiety/fear and the traumatic experiences you have been through? Are they similar, and if yes, to what extent? 4) If you got infected, do you have enough information on where to go / what to do? 5) How well informed are you about the virus? 6) Which media channels do you mainly get information from?

To recruit the refugees, the author contacted four NGOs and two housing shelters in Berlin by email. The Place4Refugees NGO and one refugee housing shelter located in Berlin Friedrichshain-Kreuzberg district agreed to help in recruiting refugees. The minimum age of participation was 18 years. The NGO’s and the shelter’s management made the introductions between the participants and the researcher. Prior to the interviews, there was no direct contact or familiarity between the author and the refugees. The main author who is proficient in Arabic conducted all the interviews by telephone, which were recorded, translated, and transcribed into English.

A total of 10 semi-structured interviews were conducted (three men and seven women). Three refugees lived in refugee accommodation shelters in Berlin (two men and one woman); the rest lived in their own apartments.

Interview transcripts were then imported to MAXQDA software. A thematic analysis was conducted in three-level coding (Hsieh & Shannon, 2005). For each interview, two members of the research team conducted the analysis to include different points of view in the analysis. In the first level, codes were formulated without any interpretation, using quotations condensed directly from the text. Each interview was analyzed independently. In the second level, the codes from the first level were abstracted and categorized based on their frequency and relatedness to each other. Finally, in the third level, the codes from the second level of all 10 interviews were combined by comparing and developing major categories. In this level, the codes were interpreted and organized into final codes.

## Results

### Sociodemographic characteristics

A total of 85 participants completed the online survey. The mean age of survey participants was 33.4 years, range 18 to 58 years; 45.9% (n  =  39) were female and 54.1% (n = 46) male. In terms of level of education, 31 participants did not answer this question. Of those who replied, 47.2% (n = 51) of the participants had a college degree, 7.4% had a technical school degree (n  =  8), 8.3% (n  =  9) reported to have obtained an upper secondary school degree, and 8.3% (*n*  =  9) stated to have no degree. The average number of persons living in a household was 3.4 (range 1 to 6); 3.6% were living alone and 28.6% were living with three persons. Ten percent (*N*  =  8) of the participants reported that they had suffered from anxiety disorder before COVID-19.

The date of migration ranged from 1986 to 2020; the greatest number of migrations (N  =  29) occurred in 2015. Seventy-seven participants provided information about their legal status in Germany: 16 (20.8%) described themselves as refugees, 37 (48.3%) were migrants (19 with permanent residence, 18 with temporary residence), 34 (31.2%) were migrants with a German passport, and three (3.9%) were in the asylum determination process.

#### COVID-19-related concerns

In terms of experiences with COVID-19, 14 participants (18%) were in quarantine at the time of answering the questionnaire; 17% (*N*  =  13) had been tested for COVID-19; and 21 (31%) said they knew someone who had tested positive.

Participants’ average rating of the risk of being infected with COVID-19 within the next month was 23.5%, which was comparable to their average rating of the risk of becoming infected with influenza (“flu”) of 21.6%.

A total of 18.2% (*n*  =  14) of the participants had not had contact with persons closer than one-meter distance outside of their household during the last week; 24.2% (*n*  =  19) reported contact with 10 or more persons. The average number of contacts outside their household was much higher for male than for female participants (*M*  =  14.34, *SD*  =  24.56 vs. *M*  =  3.43, *SD*  =  4.65, *p*  =  .01, t  =  2.42).

Participants reported an average of 186 minutes per day thinking about COVID-19. The maximum was a full day while the minimum was none; 25.0% of participants thought about COVID-19 for at least three hours per day. On average, men spent more hours per day thinking about COVID-19 (*M*  =  4.65, *SD*  =  6.11) than did women (*M*  =  1.83, *SD*  =  2.10). Those who thought for less than two hours per day about COVID-19 showed on average lower PHQ-4 scores compared to those who thought for more than two hours (*M*  =  5.6, *SD*  =  2.8 vs. *M*  =  3.46, *SD*  =  3.1, *p*  =  .04, t  =  1.59).

**Figure 1.**
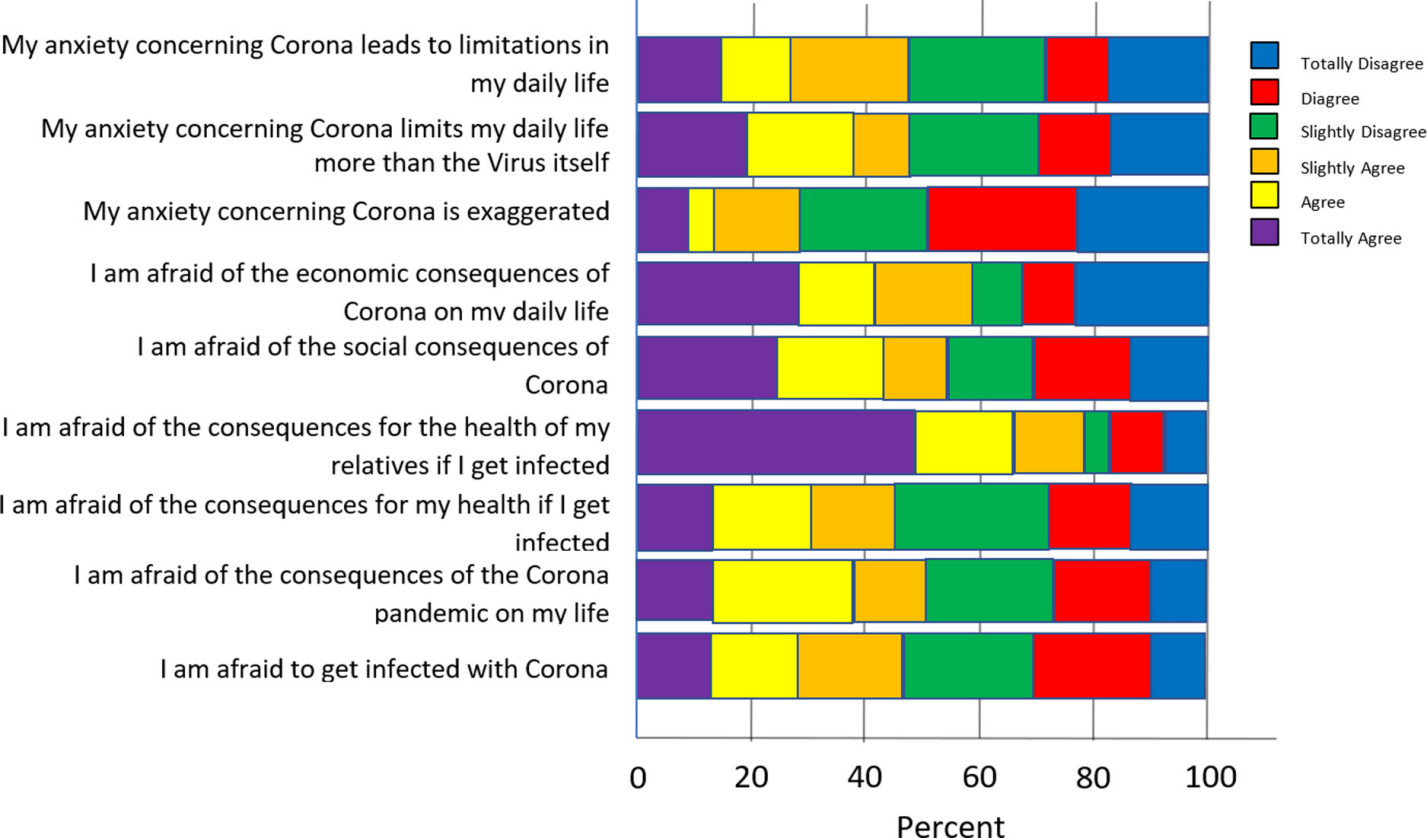
Anxiety regarding COVID-19.

 Figure 1 shows the distribution of answers to the questions about anxiety regarding COVID-19: 54.5% agreed that they were afraid of becoming infected with COVID-19; 76.4% said they were afraid of the consequences for the health of their relatives; and 56.4% reported being afraid of the economic consequences on their life. The median anxiety sum score was the highest for the refugee group ( $\tilde{x}$ =  3.5) when compared to migrants ( $\tilde{x}$ =  3.3) and holders of a German passport ( $\tilde{x}$ =  2.7). 23.2% of participants said they were afraid of death in general.

The mean PHQ-4 total score was 4.05. There was no difference in the median PHQ-4 score between men and women (*M*  =  3.82, SD  =  3.02 vs. *M*  =  4.3, SD  =  3.00); however, the range for women was wider compared to that of men (0–12 vs. 0–9).

**Figure 2.**
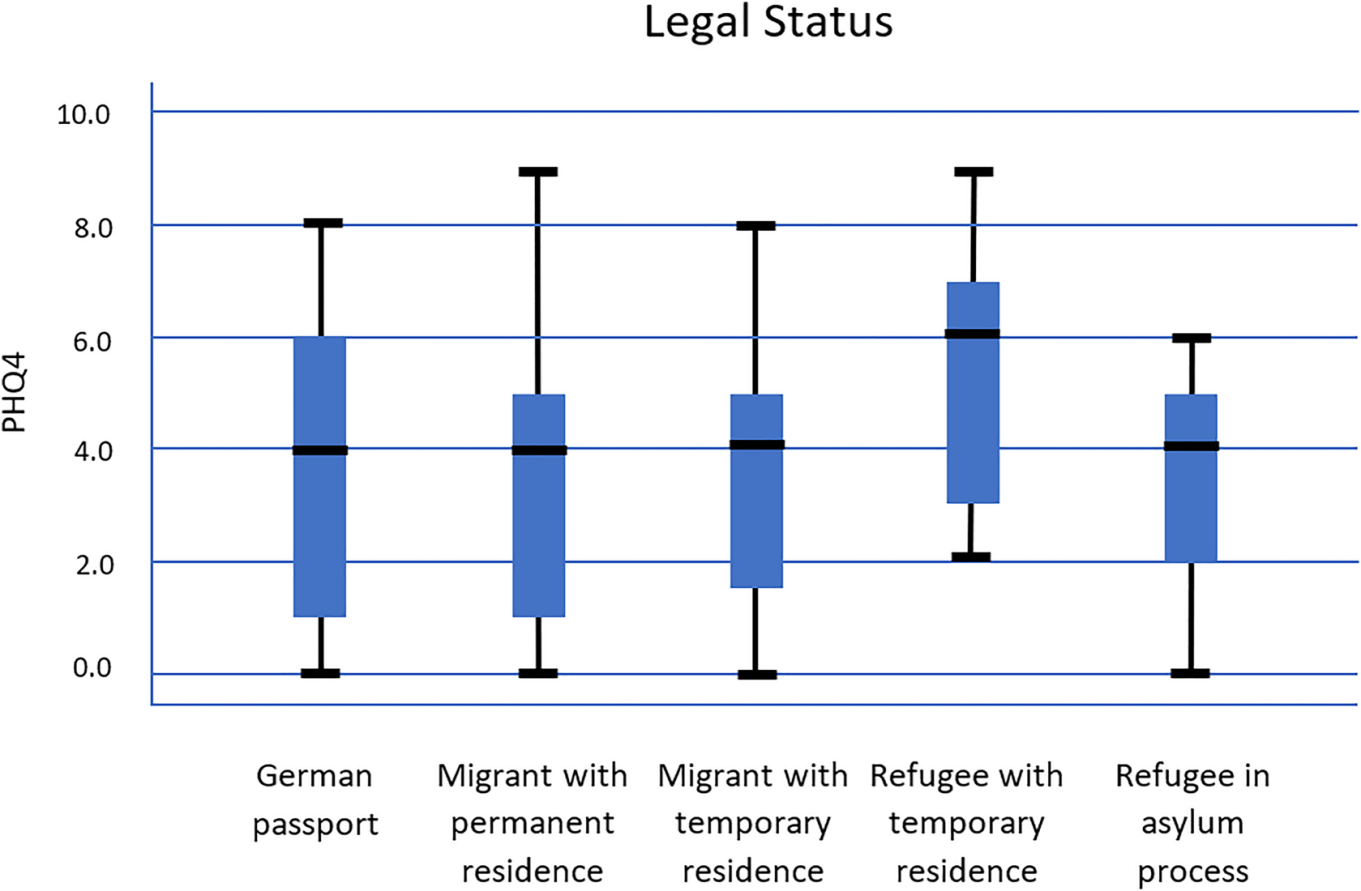
PHQ-4 score and legal status.

The median total PHQ-4 score for refugees was the highest when compared to migrants and holders of a German passport ( $\tilde{x}$ =  5.4); 25% had a PHQ-4 score of at least 6, while 25% had a PHQ-4 under 1 (Figure 2).

### Specific COVID-19 phobia symptoms

**Figure 3.**
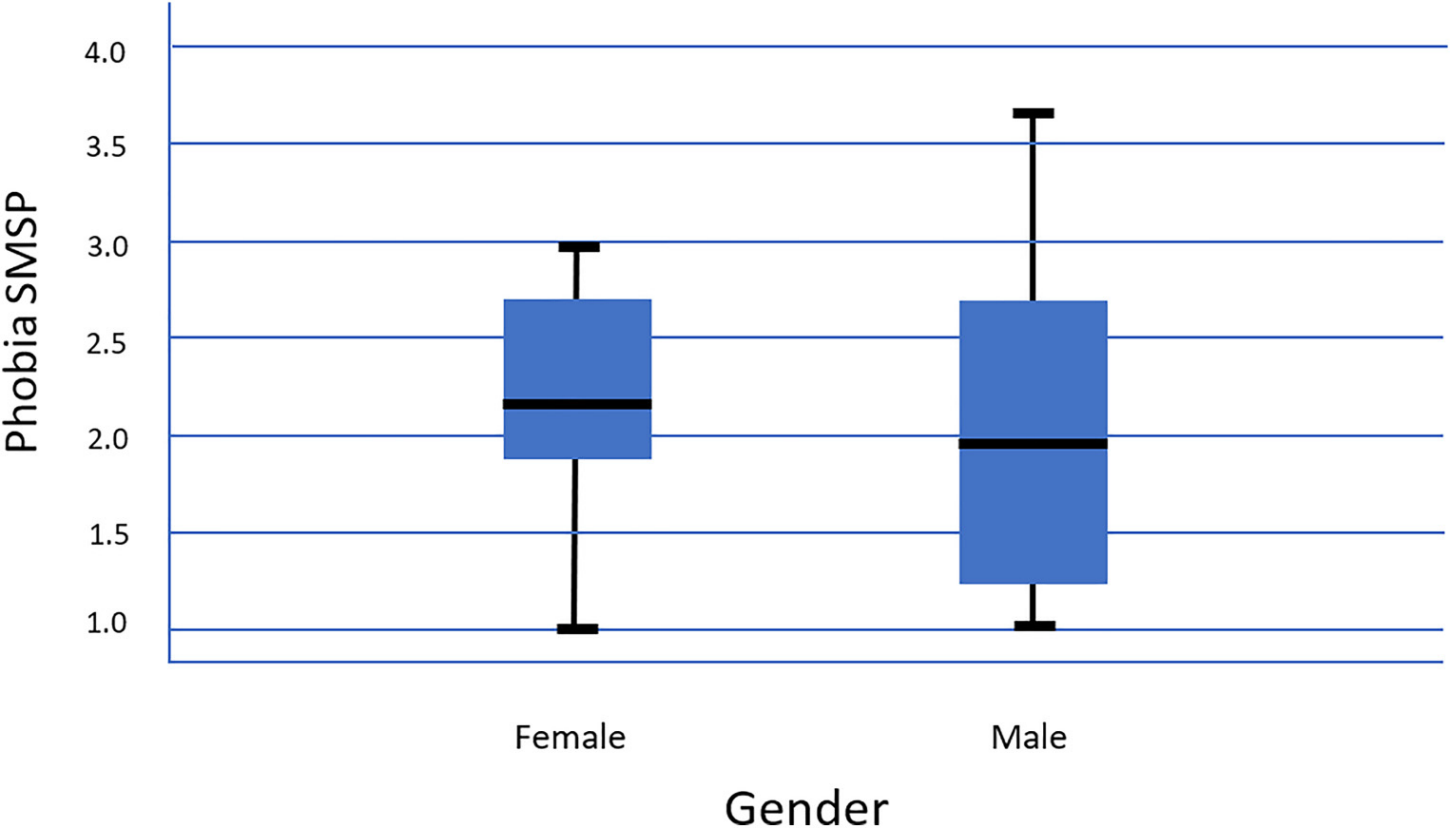
Specific COVID-19 phobia symptoms and gender.

A total of 32.7% (*N*  =  25) of participants indicated severe COVID-19-related anxiety symptoms based on the modified Specific-Phobia Scale C-19-A, with an average score above 2.5. Women had on average a slightly higher phobia level compared to men (*M*  =  2.2, SD  =  0.64 vs. *M*  =  2.0, SD  =  0.83, p  =  .05, t = 1.48); see Figure 3.

The average time spent daily on media was 98 minutes. The internet, mainly social media platforms, was the primary source of information. There were no differences regarding media hours between men and women (*M*  =  99 vs. *M*  =  100) and no significant correlations between media consumption and COVID-19-specific phobia symptoms.

### Qualitative results

This section provides an overview of the main findings generated from the interviews and classifies them into four main themes: 1) Mental health issues due to COVID-19; 2) Misconceptions about COVID-19; 3) Belief in God and other protective strategies; 4) Avoidance of an Arab neighbourhood in Berlin. In Table 1, the main themes are presented and illustrated, with sub-themes listing examples reported by the participants.

**Table 1.** Third- and second-level codes extracted from the interviews.

Main themes	Sub-themes
Mental health issues due to COVID-19	Increase of already existing depression during COVID-19 Associating everything with COVID-19 COVID-19 preventing one from living peacefully after war Postponed plans due to COVID-19 Reduced physical activity due to COVID-19 Boredom: Sitting at home and having nothing to do COVID-19 means the end of the world Worries of not seeing the family back home again due to COVID-19 COVID-19 having harder impacts on parents and their children Specific worries about children Coronavirus impacts children’s mental health, who have various questions COVID-19 fear vs. in-transit fear COVID-19 fear is similar to in-transit fear (middle stage of the relocation process); both remind of death Fear due to flight/sea during flight is stronger than COVID-19 fear
Misconceptions about COVID-19	Misconceptions “The sun / high temperature amplifies the virus” “The sun kills COVID-19, it will all end in July” Perceived low risk of infection due to age / safety measures COVID-19 is a plague Lack of information Lack of information and dependence on translators if infected Dependence on social media platforms for health information Using Facebook / social media for health information Random stories being heard by refugees on Facebook
Belief in God and other protective strategies	Engaging in religious activities to forget about COVID-19 (e.g., praying)
Avoidance of an Arab neighbourhood in Berlin	People do not seem to abide by restrictions in the Arab neighbourhood Disappointment from Arab street: high prices / no measures / quality

### Mental health issues due to COVID-19

All the participants expressed a worsening of their already existing psychological distress. One of them described it as being “the end of the world” to him. Others expressed perceiving that everything had become dependent on the COVID-19 situation. For example, the German language classes that used to keep them busy during the daytime, and the foreigners’ authorities that they require to issue their residence permits and get a sense of stability, were no longer operating: “the problem is that I had a language course, and they postponed it due to COVID-19; I have nothing to do now” (Interview 4, female); “Yeah it is boredom. You sit you sit. I also do not know the language much. Where do I go? Where do I return?” (Interview 4, female).

Refugees also expressed worries and feelings of guilt toward their families back home in Syria and Iraq. They knew that the healthcare systems there would not be able to manage the pandemic circumstances, so they ended up calling their relatives every day during the crisis: “I am worried about my mother; she is old. I heard that the virus infects old people, my uncle has diabetes also. I have the fear that I would not see them again in my life” (Interview 1, male).

Also, pandemic-related fear reminded the participants of their fear of transit when they travelled by boat to reach refuge. One described that COVID-19 did not allow her to live peacefully after war: after she made it through all the difficulties, a virus could now end her life:

Honestly, they are the same, both fears are the same. I cannot find a difference. In Syria, because of war, there was always a threat that you could die, and with this virus it is also the same. If you got infected, you could die! The same fears in Syria, same fears here due to COVID-19. (Interview 3, female)

The previous fear [fear during the flight to Germany] is different; I could see a positive ending, a new future, it was mixed feelings, fear and happiness at the same time inside me. With COVID-19 fear it is different, anything I touch I will get infected. After all I survived, a virus will end your life. I am really scared of death, but then I say your destiny will come, what is written is written, if God wanted me to die because of COVID-19 let it be. Even my fear during my life in Iraq, COVID-19 fear is bigger! With COVID-19 fear I see death. (Interview 4, female)

Parents also reported facing harder situations than participants without kids. They reported that COVID-19 affected the mental health of their children who were locked at home and asking about school and playgrounds: “the kids are depressed. You know what I mean? They want to go to school, there is no school. They want to go to kindergarten the young one, there is no kindergarten” (Interview 5, male).

### Misconceptions about COVID-19, lack of information, and dependence on social media for medical knowledge

It was clear from the interviews that refugees had several misconceptions about COVID-19. Some of them expressed that the sun would kill the virus; others that humidity increases the intensity of the virus: “The sun kills even the mold in the cellar … I think the virus will go away in July” (Interview 1, male); “I know that in summer because of the humidity, the virus will be stronger, I am not sure if this is scientific” (Interview 8, female).

All the refugees interviewed expressed a lack of information on where to go if medical help was needed. They showed dissatisfaction with the German healthcare system in general, and felt dependent on translators. Many interviewees also saw their regular medical/psychiatric appointments suspended during the pandemic, which led to more frustration:

No no I do not know, I do not have enough information. If I got infected, I would say this is the end. I will have to call the hospital I think, but first I have to get in contact with the translator as I cannot tell them what I feel. (Interview 4, female)

There is no communication here between the patient and the physician. He even told me to see an Arabic-speaking doctor instead. (Interview 1, male)

All participants reported using social media channels to obtain information about COVID-19, mainly Facebook and YouTube channels: “Well I get the information I need from the media; the phones and you know Facebook also” (Interview 10, male).

### Belief in God and other protective strategies

When asked about protective strategies they use to handle the COVID-19 situation, all participants expressed that their belief in God is the only thing that puts their mind at rest: “we count on God, that if we abide by the safety measures, nothing will happen” (Interview 3, female); “They say this disease kills. Listen, death is in the hands of God Almighty. As you know we Muslims, we believe that no one dies until his time is over” (Interview 5, male).

In addition, one male participant stated that he started going fishing during COVID-19 to forget about the virus, and one female participant noted that she started cooking more complicated Middle Eastern dishes in order to forget a bit about COVID-19.

However, not all of them were able to engage in activities to soothe their mental health. One female participant was continuously experiencing racism in her neighbourhood, and this hindered her from going out. She was in continuous quarantine due to the racism she experienced in her everyday life and due to the pandemic:

I am imprisoned because of COVID-19 and racism at the same time. My immune system is not that strong; you know yesterday I did a blood test, and I fainted. My social worker told me you can still go out have a walk during COVID-19, you should forget about racism. (Interview 4, female)

### Avoidance of an Arab neighbourhood in Berlin

Although it was not part of the questionnaire, the topic of an Arabic district in Berlin popped up in the first four interviews. The Arab district—mainly called the “the Arab street” (Sonnenallee)—in Berlin shows the effects of migration, and particularly of the more recent refugee migration after the war in Syria. This area has been changed due to reshaping and diversification processes of the physical and social spaces of the street and is now mainly dominated by Syrians (Steigemann, 2020). Participants primarily explain that the reason for not abiding by the preventive measures in this street is religion. One interviewee described that religious people think that God will protect them against sickness, and that whatever God has written will happen:

Well, [in the Arabic neighbourhood] they do not abide by the rules. I hear a lot of people saying God will protect us, whatever you do, and whatever preventions you take, what is written will come to you. This is a pandemic, the virus does not differentiate between religions, people, or colour. (Interview 4, female)

At [a German supermarket] they put a limited number of carts out to reduce traffic inside the supermarket, they disinfect what goes in and out. Everyone entering and leaving gets their hands disinfected. In the Arab neighbourhood it is not the case, the carry carts are between the hands of all the people. Whoever wants to carry a cart can carry one, or whoever wants to walk in and touch the products and leave them standing. It is still suffocating, I avoid going there. (Interview 2, male)

## Discussion

This article explored how the current COVID-19 pandemic is connected to a psychological burden among migrants and refugees in Germany.

In our sample, the mean PHQ-4 total score was 4.05 and in the German-speaking sample was 4.15 (Petzold et al., 2020), both of which are higher than the general population before the COVID-19 pandemic (*M*  =  1.76) (Petzold et al., 2020). This could show that due to the current crisis, there is an increase in depressive and anxiety symptoms (Petzold et al., 2020). The fact that the PHQ-4 scores were slightly lower in our sample than in the German general population (average PHQ-4 of 4.15) could be because Germany is considered an individualistic country compared to Arab countries (67 vs. 38 Individualism Index Score) (Hofstede, 2011). In individualistic societies, people are expected mainly to look after themselves and their immediate family; in contrast, in collectivistic societies, people tend to see themselves as belonging ‘in groups’ that take care of them, which may provide more social support and help reduce loneliness during the lockdown. This was clearly demonstrated in the interviews, where all the interviewees reported that they had more frequent contact with their relatives and families in Syria as well as in Germany during COVID-19. Another reason might be related to their war experiences, as one interviewee reported that he had witnessed severe situations during the war in Syria that were more traumatizing than COVID-19. The median total PHQ-4 score for refugees was the highest when compared to the migrants who have a residence permit and who have a German passport. This could be explained by the instability in their residence status and the fear of deportation (Dalexis & Cénat, 2020).

Overall, about half of the participants feared becoming infected with COVID-19, and more than half (76.4%) were afraid of the consequences for the health of their relatives. The median anxiety score was again highest for the refugee group. This is comparable with the German-speaking population, where social consequences seem to be a more salient concern than economic consequences (Petzold et al., 2020).

We also found that the participants reported an average of 186 minutes per day thinking about COVID-19; this is less than what was found in another study in the German general population (285 minutes on average per day) (Petzold et al., 2020). Interestingly, and unlike the results in many other studies, men in our sample spent on average more hours per day than women thinking about COVID-19. This might be attributed to several reasons, such as the fact that Middle Eastern men usually tend not to display feelings and worries or seek social support as much as Middle Eastern women do, but rather face the matter internally and therefore think longer about it. In addition, Middle Eastern women tend to be busy with the care of children and have less time to think about COVID-19, or also as shown in our results, men were going out more often than women and thus had higher chances to get infected which may have led them to worry more. Only one study (Ozamiz-Etxebarria et al., 2020) indicated that men have higher levels of depression than women in their sample, but similar levels of anxiety and stress (Ozamiz-Etxebarria et al., 2020).

The average time spent daily on media was 98 minutes, less than for the German-speaking population (144 minutes) (Bendau et al., 2020). Social media platforms were the main source of information, whereas it was official websites of the government or health authorities for the German-speaking population (Bendau et al., 2020). In our sample, we did not find a significant correlation between media use and COVID-19-related anxiety symptoms. Bendau et al. showed that participants who reported social media as a primary source of information showed—in comparison to participants not using social media—on average significantly more unspecific anxiety and depression and significantly more specific COVID-19-related anxiety symptoms (Bendau et al., 2020). Therefore, it could be relevant that social media was a primary source of information for many of the assessed migrants and refugees. During the pandemic, it is recommended to moderate media consumption so that one can stay informed but avoid excess or incorrect information (Wiederhold, 2020).

The average rating of the risk of becoming infected with COVID-19 within the next month was comparable with the risk of getting infected with influenza, whereas in the general population the risk was seen as more than double the size for COVID-19 compared to influenza (Petzold et al., 2020). The reported risk of becoming infected with influenza in April (21.6%) seems quite high, as influenza is seasonal. This similarity in the risks between influenza and COVID-19 might be due to the excessive information shared on social media platforms that claimed that both diseases are contagious and have very similar symptoms, which created an association between both illnesses, irrespective of the season or the time of year. Another reason for this similarity might be due to a lack of awareness of the fact that influenza is seasonal.

Men tended to have more contacts outside their household than women. This could be explained by the role of men in being the providers and breadwinners of the family (Asiazobor et al., 2016). This is also demonstrated in the qualitative interviews where, for example, it was the male interviewees going out for the grocery shopping during the pandemic and not the female interviewees.

Results from the qualitative interviews show that the COVID-19 pandemic and its consequences increased the pre-existing psychological burden of refugees specifically and reminded them of the war in their home countries and their flight experiences. This is consistent with the observations of Srivatsa and Stewart (2020) that Ebola virus survivors are at high risk for mental health problems (post-traumatic stress and other anxiety disorders and mood disorders).

Refugees also had no information on where to get medical help if they became infected, and were very much dependent on translators. Therefore, effective communication and information should be available, as immigrant populations are often vulnerable to severe health disparities due to a lack of adequate health communication that helps them recognize, minimize, and respond to health-related problems (Kreps & Sparks, 2008). Pandemic communications should create an inclusive atmosphere within the population and include patients/population as effective partners by encouraging them to contain the pandemic, and foster resilience and protective factors (Vaughan & Tinker, 2009).

Refugees indicated many existing communication barriers between them and the German healthcare providers in general; therefore, culturally sensitive communication, outreach activities, and self-help interventions during pandemics would help difficult-to-access urban populations, undocumented immigrants, and non-German-speaking refugees (Vaughan & Tinker, 2009).

According to the American Psychological Association (2020), during periods of social distancing, quarantine, or isolation, people are more likely to experience fear and anxiety; depression and boredom; anger, frustration and irritability; and stigmatization—especially people with pre-existing mental health issues and healthcare workers (American Psychiatric Association, 2020). This is in parallel with our results. All respondents reported that their main difficulties during quarantine for COVID-19 were increased levels of boredom and loneliness. Before COVID-19, they had busy schedules, attending German classes, taking their children to kindergarten, and attending various appointments at the foreign office. However, this changed during quarantine; their days became emptier. Loneliness and social isolation from the community may contribute to heightened distress during quarantine (Vaughan & Tinker, 2009). Interventions that can safely enhance social connection while maintaining social distance, such as video conferences, are necessary. Also, Xiao (2020) suggests “structured letter therapy” (remote written counseling) and telehealth as options to continue counseling during the pandemic (Xiao & Wang, 2020).

In our study, respondents did not express an increase in alcohol or drug intake as a response to stress and isolation. The main strategy they used to handle the situation was praying and having faith that God would prevent infection. In general, during this period of isolation a spike in alcohol misuse and development of alcohol use disorders among the general population was expected (Clay & Parker, 2020).

As for the refugees, although religiosity helped them to better handle the psychological burden of the pandemic, their socioeconomic status might have prevented them from abiding by the safety measures recommended by the government and the WHO, such as avoiding public transport and overcrowded spaces, and working from home when possible. Many refugees still live in overcrowded refugee housing and have to go to work and risk their lives to earn a living (Dalexis & Cénat, 2020). One of our interviewees mentioned that he was a cleaner and had to go to work every day. These behaviors were the result of their low socioeconomic status and were sometimes justified with their fatalistic views that God is the protector. Thus, pandemic-mitigating interventions should consider cultural competency and connections between religious/cultural beliefs, socioeconomic factors, and health practices that are critical in the success of public health interventions targeting the refugee and migrant population (Truman et al., 2009; Vaughan & Tinker, 2009).

The main sources of information were internet platforms, mainly Facebook and YouTube channels, which can propagate both true and false theories and information, making it hard to distinguish one from the other. Some refugees believed that the sun would kill the virus; others believed that humidity would do so—however, the COVID-19 virus can spread in hot and humid climates, and likewise, cold weather and snow cannot kill the virus (WHO, 2020d). Mian and Khan (2020) argue that regarding COVID-19, misinformation has spread far and wide, drowning out credible sources of information (Mian & Khan, 2020).

## Strengths and limitations

This study represents the first mixed-methods study that assesses psychological distress, anxiety, and depression in the current COVID-19 pandemic among refugees and migrants in Germany.

However, there are some limitations. The study design is cross-sectional, therefore drawing conclusions about causality is not possible. The sample was recruited as a convenience sample mainly through social media (Facebook and WhatsApp), which might have led to a sample bias. Also, it included only Arabic-speaking participants; therefore we cannot make reliable conclusions about other refugees or migrant groups. In addition, the sample was small for some subgroups; thus no adequate comparison of different subgroups was possible.

In the quantitative part, people who are familiar with social media might have been more likely to be selected. In the qualitative part, only refugees connected to the Place4Refugees NGO and the housing shelter in Berlin—who agreed to help in recruiting—were interviewed. Also, in the interviews, religiosity was not assessed, although it could have provided additional information about the sample.

## Conclusion

The rapid spread of COVID-19 tells us how we are all interconnected in terms of our health and well-being. Refugees need to have enough information regarding access to the mental health system in Germany and reliable information about COVID-19 from trusted sources rather than social media. The use of mistrusted sources from the internet is a risk to public health. Governments should develop strategies to regulate health information on the internet, especially for the refugee population.

The need for inclusive national public health measures is strongly emphasized. Refugees and migrants, irrespective of their legal status, must be provided with access to healthcare, other services, and culturally and linguistically sensitive information campaigns on how to prevent being infected and infecting others, and must consider social determinants such as discrimination and criminalization in their response operations (WHO, 2020a).

More targeted culturally sensitive interventions that concentrate on vulnerable populations are needed. Smartphone-based behaviour therapy, self-help, and self-care interventions should focus on relaxation exercises. Efforts to develop effective treatments and vaccines should be coupled with strategies to support the psychological needs of the public overall, as well as of infected patients and of vulnerable populations such as refugees and migrants.

Recommended strategies to reduce psychological distress in the pandemic such as a healthy lifestyle, maintaining virtual social networks, acceptance of negative emotions, and avoidance of rumours and myths are supported by our data.
